# Correlation between protein kinase catalytic subunit alpha-1 gene rs13361707 polymorphism and gastric cancer susceptibility in asian populations

**DOI:** 10.18632/oncotarget.19355

**Published:** 2017-07-18

**Authors:** Jianfeng Ni, Nan Shen, Jilei Tang, Kewei Ren

**Affiliations:** ^1^ Department of Gastroenterology, Tongzhou People’s Hospital of Nantong, Nantong 226300, China; ^2^ Department of Clinical Pharmacy, The Affiliated Jiangyin Hospital of Southeast University Medical School, Jiangyin 214400, China; ^3^ Department of Orthopedics, Qidong People’s Hospital, Nantong 226200, China; ^4^ Department of Orthopedics, The Affiliated Jiangyin Hospital of Southeast University Medical School, Jiangyin 214400, China

**Keywords:** protein kinase catalytic subunit alpha-1, gastric cancer, polymorphism, risk, meta-analysis

## Abstract

A single nucleotide polymorphism (SNP) of the protein kinase catalytic subunit alpha-1 gene (*PRKAA1*) that confers susceptibility to gastric cancer (GC) was identified by genome-wide association in several case-control studies. However, the results remained controversial and ambiguous. Therefore, we performed a larger meta-analysis to confirm this association. We searched the PubMed, Embase, WanFang, and CNKI databases, without any restriction on language, covering all papers published until Feb 22, 2017. Overall, 14 case-control studies with 14,485 cases and 14,792 controls were retrieved based on the search criteria. Odds ratios (ORs) with 95% confidence intervals (CIs) were used to quantify the strength of the association. Publication bias was assessed by Egger’s and Begg’s tests. We found that the *PRKAA1 rs13361707* C/T polymorphism had no association with GC risk in any of the pooled genetic models (for example, the T-allele vs. C-allele allelic contrast model yielded the following estimates: OR = 0.87, 95% CI = 0.73–1.05, *P*_heterogeneity_ = 0.000). Furthermore, in analyses stratified by either source of control or geographical origin of subjects, a statistically significant inverse relationship was detected between *PRKAA1 rs13361707* C/T polymorphism and GC risk. No obvious evidence of publication bias was detected in the pooled meta-analysis. Furthermore, we observed that individuals carrying T-allele (TT or TC) genotypes had a lower expression of *PRKAA1*. Our present study indicated that *PRKAA1 rs13361707* C/T was not significantly associated with GC risk, despite few positive results in the subgroups.

## INTRODUCTION

Gastric cancer (GC), including both the cardia and non-cardia types, is the second leading cause of cancer mortality in the world, with an estimated 720,000 gastric cancer-related deaths in 2012 [1]. Among various population groups of the world, the highest incidence and mortality of gastric cancer were registered in Northeast Asian countries (China, Japan, and Korea) accounting for more than half of the global figures [2, 3]. Indeed, the incidence of and mortality from gastric cancer in these countries rank the highest among digestive tract cancers.

The epidemiology of GC is complex and multifactorial in nature. Several environmental factors, including alcohol consumption, diet, tobacco smoke, exposure to *Helicobacter pylori* (*H. pylori*), and a history of stomach disorders have been considered potential risk factors [4]. Additionally, genetic susceptibility may also contribute to GC risk [5, 6].

In 2011, Shi et al [7]. first performed a genome-wide association study (GWAS) of non-cardia GC, employing a case-control design on subjects of Han Chinese descent, by genotyping 906,703 SNPs. They discovered that the protein kinase catalytic subunit alpha-1 gene *(PRKAA1) rs13361707* polymorphism was significantly associated with GC risk (OR = 1.42, 95% CI = 1.24–1.62, *P* < 0.01).

PRKAA1 is also known as AMPKa1. It is the catalytic subunit of 5’-AMP-activated protein kinase (AMPK), which is a cellular energy sensor conserved across all eukaryotic cells. The kinase activity of AMPK is activated by stimuli that increase the cellular AMP/ATP ratio. AMPK regulates the activities of a number of key metabolic enzymes through phosphorylation. It protects cells from stresses that cause ATP depletion by switching off ATP-consuming biosynthetic pathways. Alternatively-spliced transcript variants encoding distinct isoforms of the protein have been observed in several diseases, including cancer [8–10].

The *rs13361707* SNP is located in the first intron of *PRKAA1* at the 5p13.1 site, which is located close to the gene for prostaglandin E receptor 4 (PTGER4). PTGER4 is a prostaglandin (PG) E2 receptor, which is a major product of cyclooxygenase-2 (COX-2) action that plays an important role in immune response and cancer pathogenesis [7]. So far, a number studies have reported that *PRKAA1 rs13361707 C/T* polymorphism is associated with gastric, colon, and rectal cancers (especially gastric cancer). However, the direction of this association remains ambiguous and the polymorphism may even be inversely related to GC risk.

In 2015, Qiu et al [11]. conducted a meta-analysis on *PRKAA1 rs13361707 C/T* polymorphism and GC risk. However, two limitations affected their study: the samples included were relatively small, and several case-control studies, including two written in Chinese, were not identified for inclusion. Subsequent to that publication, even more case-control studies have been published [12–14]. Therefore, to ascertain the precise association between the *PRKAA1 rs13361707 C/T* polymorphism and GC risk, especially in the light of new publications, an updated meta-analysis of all eligible case-control studies [7, 11–19] was necessary. On conducting an updated meta-analysis, we found a few results contradictory to the conclusions of Qiu et al.

## RESULTS

### Eligible studies

A total of 51 articles were retrieved based on our selection strategy from the PubMed, Embase, WanFang, and CNKI databases. Among them, 33 articles were excluded because they did not provide information about the *PRKAA1* polymorphism. Another eight duplicated articles were also excluded. The publication by Shi et al. [7] included five different case-control sub-studies, all of which were also adopted into our study, although overlapping or duplicated data may exist among them. Thus, a total of 10 articles about 14 case-control studies with 14,458 cases and 14,792 controls were included in our meta-analysis [7, 11–19] (Table 1). The 14 studies could be stratified according to the following independent criteria: (a) Source of controls: six were hospital-based (HB) and eight were population-based (PB); (b) Country: 11 were from China and three were from Korea; (c) Size of the case and control groups: in 9 studies, less than 1000 samples were included, whereas there were more than 1000 samples in 5 studies.

**Table 1:** Study characteristics from previous published studies on the association between *PRKAA1 gene* rs13361707 polymorphism and GC risk

Author	Year	Origin	Design	Subjects size	Case	Control	Case			Control				Method	GC type	NOS
							TT	TC	CC	TT	TC	CC	HWE			
Eom	2016	Korea	HB	<1000	846	846	176	421	249	248	424	174	0.77	GoldenGate assay (Illumina)	NA	6
Zhang	2016	China	HB	<1000	60	60	10	27	23	16	34	10	0.26	MALDI-TOF	NA	8
Kim	2014	Korea	HB	<1000	475	473	97	241	137	135	242	96	0.51	GoldenGate assay (Illumina)	non-cardia	7
Wu	2014	China	HB	<1000	217	428	48	115	54	133	209	86	0.81	Multiplex SNaPshot SNP	NA	6
Li	2013	China	HB	<1000	335	334	71	167	97	102	165	67	0.98	TaqMan	NA	7
Dong	2015	China	HB	<1000	167	186	62	68	37	41	91	54	0.82	iMLDR	NA	8
Song	2013	Korea	PB	>1000	3245	1700	682	1654	909	477	846	377	0.95	HRM-PCR	non-cardia	8
Qiu	2015	China	PB	>1000	1124	1194	209	571	344	356	565	273	0.09	TaqMan	cardia	8
Shi	2011	China	PB	<1000	979	2268	302	517	160	507	1154	607	0.35	AGWHSA 6.0 chips	non-cardia	8
			PB	>1000	1873	2076	561	941	371	464	1034	578	0.97		non-cardia	8
			PB	>1000	1392	1513	480	675	237	376	745	392	0.56		non-cardia	8
			PB	<1000	895	3227	225	447	223	898	1616	713	0.78		NA	8
			PB	>1000	2404	3227	459	1221	724	898	1616	713	0.78		NA	7
Cai	2017	China	PB	<1000	473	487	88	213	172	143	246	98	0.67	KASP	NA	6

PB: population-based; HB: hospital-based; MALDI-TOF-MS: polymerase chain reaction–matrix-assisted laser desorption/ionization time-of-flight mass spectrometry; HRM-PCR: high resolution melting-polymerase chain reaction; iMLDR: improved multiplex ligase detection reaction; AGWHSA 6.0 CHIPS: Affymetrix Genome-Wide Human SNP Array 6.4 chips; KASP: kompetitive allele-specific PCR; HWE: Hardy–Weinberg equilibrium of control group; NA: not available information.

We also checked the MAF (minor-allele frequency; the frequency of the most common mutant allele) of the *PRKAA1 rs13361707 C/T* polymorphism among the five main population groups reported in the 1000 Genomes Browser: East Asian (0.55), European (0.29), African (0.36), American (0.25), and South Asian (0.33). The MAFs in our analysis were found to be 0.49 and 0.51 for the case and control groups, respectively. Our estimates accorded well with the East Asian MAF reported in the 1000 Genomes Browser database. Finally, the distribution of genotypes among controls was consistent with Hardy–Weinberg equilibrium (HWE) in all models.

### Meta-analysis

**Figure 1:**
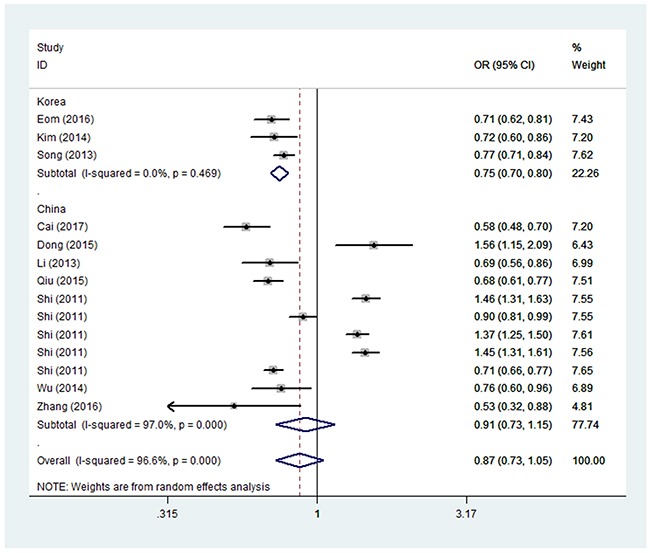
Forest plot of GC risk associated with *PRKAA1 rs13361707 C/T* polymorphism (T-allele vs. C-allele) in the whole The squares and horizontal lines correspond to the study-specific OR and 95% CI. The area of the squares reflects the weight (inverse of the variance). The diamond represents the summary OR and 95% CI.

In the pooled analysis of all Asian populations, no association could be observed between GC risk and the genotypic variants of *PRKAA1 rs13361707 C/T*. The different genetic models of polymorphism-associated risk tested for the pooled Asian population were: allelic contrast (OR = 0.87, 95% CI = 0.73–1.05, *P*_heterogeneity_ < 0.01, Figure 1), homozygote comparison (OR = 0.76, 95% CI =0.53–1.10, *P*_heterogeneity_ < 0.01), heterozygote comparison (OR = 0.88, 95% CI = 0.72–1.07, *P*_heterogeneity_ < 0.01), the dominant allele model (OR = 0.83, 95% CI = 0.65–1.07, *P*_heterogeneity_ < 0.01) and the recessive allele model (OR = 0.84, 95% CI = 0.65–1.07, *P*_heterogeneity_ < 0.01). The lack of association in our models is diametrically opposed to the conclusions reported by Qiu et al. [11] (Table 2).

**Table 2:** Total and stratified analysis of *PRKAA1 gene* rs13361707 polymorphism and GC risk

Variables	N^a^	Case/Control	T-allele vs. C-allele			TC vs. CC			TT vs. CC			TT+TC vs. CC			TT vs. TC+CC		
			OR (95%CI)	*P*_h_^b^	*P^c^*	OR (95%CI)	*P*_h_	*P*	OR (95%CI)	*P*_h_	*P*	OR (95%CI)	*P*_h_	*P*	OR (95%CI)	*P*_h_	*P*
Total	14	14485/14792	0.87(0.73-1.05)	0.000	0.140	0.88(0.72-1.07)	0.000	0.193	0.76(0.53-1.10)	0.000	0.142	0.83(0.65-1.07)	0.000	0.155	0.84(0.65-1.07)	0.000	0.160
Source of Control																	
HB	6	2100/2327	0.78(0.63-0.97)	0.000	0.025	0.73(0.63-0.84)	0.306	0.000	0.61(0.40-0.92)	0.306	0.020	0.69(0.54-0.89)	0.018	0.004	0.75(0.54-1.04)	0.306	0.085
PB	8	12385/12465	0.93(0.73-1.19)	0.000	0.586	0.97(0.75-1.26)	0.000	0.873	0.88(0.54-1.44)	0.000	0.614	0.94(0.68-1.31)	0.000	0.731	0.89(0.65-1.24)	0.000	0.501
Country																	
China	11	9752/11587	0.91(0.73-1.15)	0.000	0.436	0.92(0.72-1.17)	0.000	0.505	0.84(0.53-1.32)	0.000	0.445	0.89(0.65-1.21)	0.000	0.456	0.90(0.66-1.22)	0.000	0.490
Korea	3	4566/3019	0.75(0.70-0.80)	0.469	0.000	0.77(0.68-0.86)	0.451	0.000	0.56(0.49-0.64)	0.469	0.000	0.69(0.62-0.77)	0.376	0.000	0.66(0.60-0.74)	0.830	0.000
Subjects scope																	
<1000	9	4447/8309	0.83(0.65-1.06)	0.000	0.131	0.79(0.59-1.07)	0.000	0.125	0.69(0.42-1.12)	0.000	0.134	0.75(0.53-1.07)	0.000	0.119	0.81(0.60-1.10)	0.000	0.183
>1000	5	10038/9710	0.94(0.69-1.29)	0.000	0.719	1.00(0.75-1.35)	0.000	0.983	0.89(0.47-1.67)	0.000	0.713	0.97(0.65-1.45)	0.000	0.881	0.88(0.57-1.37)	0.000	0.573
GC type																	
NA	8	6775/5522	0.71(0.70-0.75)	0.149	0.000	0.71(0.62-0.82)	0.079	0.000	0.51(0.46-0.57)	0.224	0.000	0.63(0.55-0.72)	0.064	0.000	0.62(0.57-0.68)	0.693	0.000
non-cardia	5	6815/9270	1.26(0.89-1.77)	0.000	0.190	1.24(0.86-1.80)	0.000	0.243	1.57(0.78-3.15)	0.000	0.205	1.36(0.84-2.19)	0.000	0.210	1.34(0.85-2.10)	0.000	0.203
cardia	1	895/3227	0.90(0.81-0.99)	-	0.140	0.88(0.74-1.06)	-	0.189	0.80(0.65-0.99)	-	0.038	0.85(0.72-1.02)	-	0.075	0.87(0.73-1.03)	-	0.110

^a^ Number of comparisons, ^b^ *P* value of *Q*-test for heterogeneity test, ^c^*P* value of *Z*-test for statistical significance, NA: not available information.

**Figure 2:**
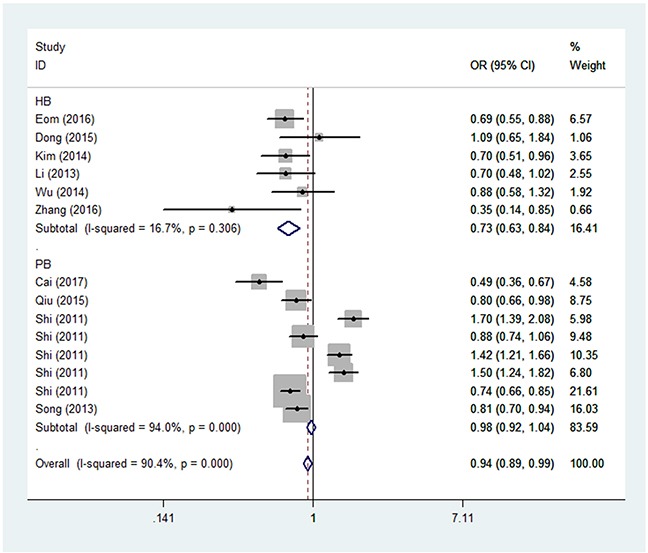
Forest plot of GC risk associated with *PRKAA1 rs13361707 C/T* polymorphism (TC vs. CC) by source of control The squares and horizontal lines correspond to the study-specific OR and 95% CI. The area of the squares reflects the weight (inverse of the variance). The diamond represents the summary OR and 95% CI.

**Figure 3:**
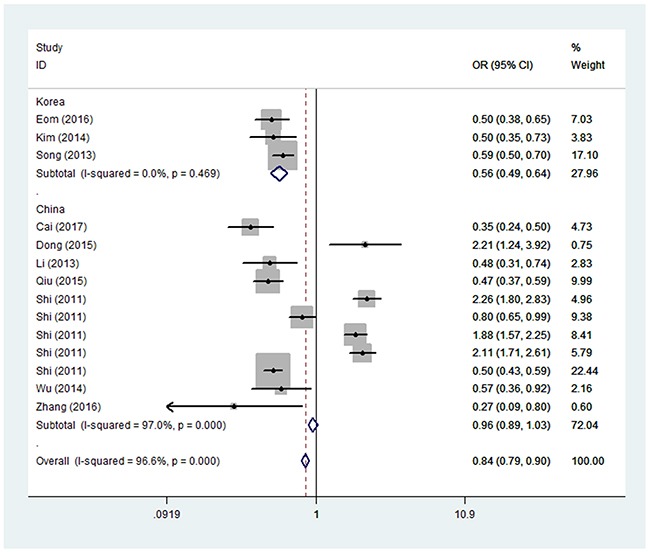
Forest plot of GC risk associated with *PRKAA1 rs13361707 C/T* polymorphism (TT vs. CC) by source of country The squares and horizontal lines correspond to the study-specific OR and 95% CI. The area of the squares reflects the weight (inverse of the variance). The diamond represents the summary OR and 95% CI. Each point represents a separate study for the indicated association. Log [OR], natural logarithm of OR. Horizontal line, mean effect size.

In the analysis stratified by source of control, significantly decreased associations were found between *PRKAA1 rs13361707 C/T* polymorphism and GC risk in the HB group (for example, the heterozygote comparison model [OR = 0.73, 95% CI = 0.63–0.84, *P*_heterogeneity_ = 0.306, *P* = 0.000] in Figure 2). In the analysis stratified by country, similarly significant associations were detected between *PRKAA1 rs13361707 C/T* polymorphism and GC risk in the Korean population (for example, in the homozygote comparison model [OR = 0.56, 95% CI = 0.49–0.64, *P*_heterogeneity_ = 0.469, *P* = 0.000] in Table 2; Figure 3). To our surprise, no association was found in the analysis stratified by sample size (Table 2).

In the analysis stratified by GC type, a similar magnitude of association was observed between *PRKAA1 rs13361707 C/T* polymorphism and GC risk in the group for which no information was available (for example, the dominant genetic model [OR = 0.63, 95% CI = 0.55–0.72, *P*_heterogeneity_ = 0.064, *P* = 0.000] in Table 2). However, no significant associations were found for non-cardia GC.

### Sensitivity analysis and publication bias

Sensitivity analysis was performed to assess the influence of each individual study on the pooled OR by sequential removal of individual studies. The results suggested that no individual study affected the overall OR significantly (Supplementary Figure 1). This suggests that our conclusion is credible and generalizable. Begg’s funnel plot and Egger’s test were performed to assess publication bias. As shown in Supplementary Table 1, the shapes of the funnel plots did not reveal an obvious asymmetry in any of the comparison models. Similarly, neither of the above tests provided any evidence of publication bias (for example, T-allele vs. C-allele, *t* = −0.49, *P* = 0.635 for Egger’s test; and z = 0.44, *P* = 0.661 for Begg’s test; Supplementary Figures 2 and 3, respectively).

### In-silico analysis of PRKAA1 expression

**Figure 4:**
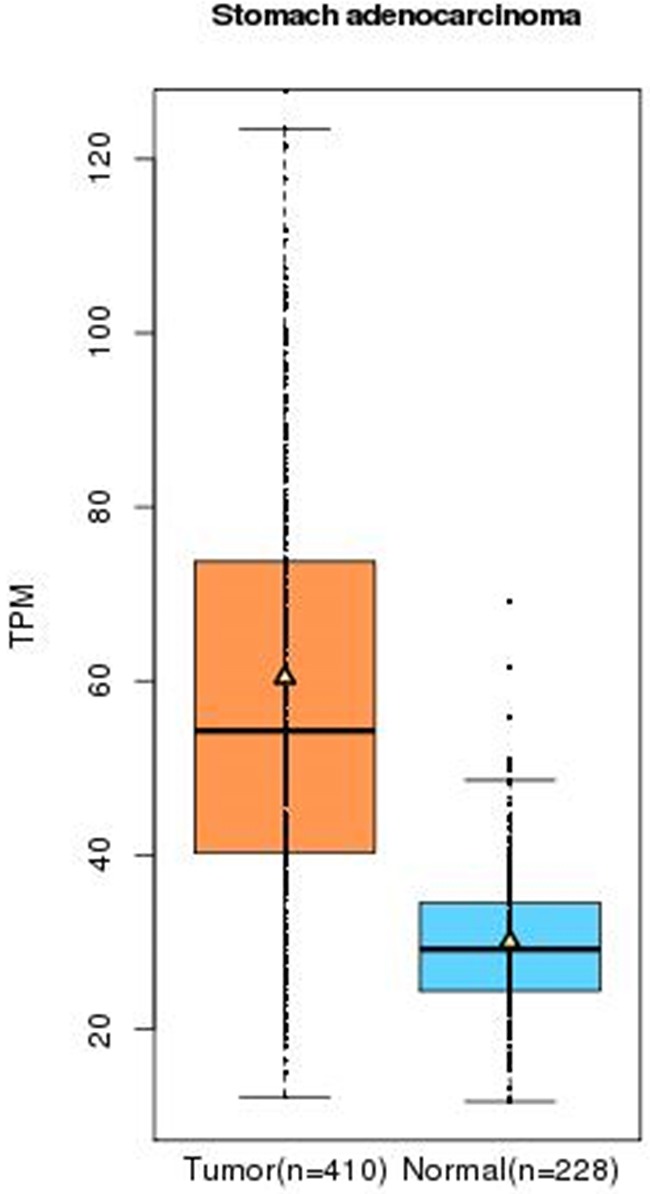
Analysis of serum *PRKAA1* levels in three genotypes of GC cases with mean values (horizontal lines, mean values) *P* = 0.031 compared with the TT/TC and CC genotypes.

In-silico results indicated that the expression of *PRKAA1* in GC tissue was higher than that in paracancerous tissue (TPM: Transcripts Per Kilobase Million = 55 vs. 30 respectively, *P* < 0.01, Figure 4).

### Serum expression of *PRKAA1* with different genotypes in GC patients

**Figure 5:**
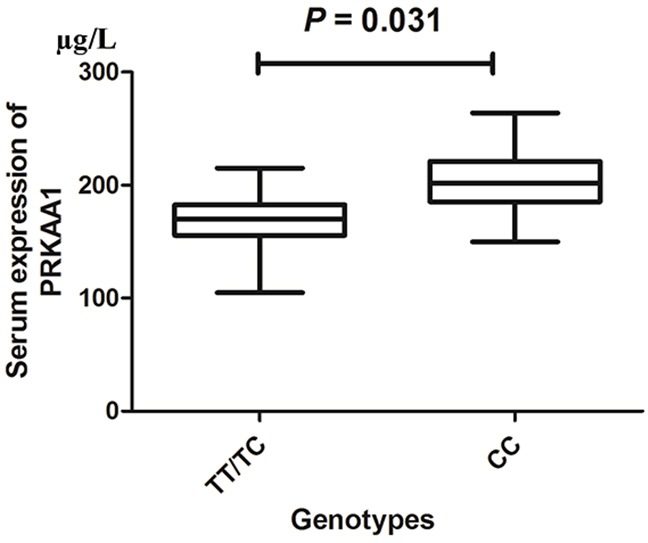
The relative expression of *PRKAA1* in GC tissue and paracancerous tissue (Normal) using TCGA database TPM (Transcripts Per Kilobase Million) stands for the expression of *PRKAA1* in each tissue.

We collected 200 serum samples of GC patients in the present study with different genotypes of the *PRKAA1 rs13361707 C/T* polymorphism. The distribution observed for the TT, TC, and CC genotypes was 40 (20%), 64 (32%) and 96 (48%), respectively. Moreover, serum *PRKAA1* levels in GC patients with TT/TC genotypes were significantly lower than in those with CC genotypes (171.18 ± 76.58 μg/L vs. 209.13 ± 51.65 μg/L, *P* = 0.031; Figure 5).

## DISCUSSION

GC remains a high-incidence malignancy but the incidence greatly varies between countries. The majority of cases are registered in developing countries, with half of them reported in Eastern Asia. In addition, GC incidence is twice as high in men as in women. This suggests that environmental, hormonal, or genetic factors may affect its risk [3]. Therefore, we selected a single nucleotide polymorphism named *PRKAA1 rs13361707 C/T* to analyze the susceptibility of Asian individuals to GC.

To evaluate the association between *PRKAA1 rs13361707 C/T* polymorphism and GC risk, we updated a previously published analysis [11] with additional publications, without excluding any relevant ones, to the best of our knowledge. We performed a meta-analysis involving 14,485 GC cases and 14,792 controls; all samples were taken from the Northeast Asian population. The power of our meta-analysis is 0.997, which suggests that our data are adequate for detecting even small effect sizes. In the pooled analysis, no association was observed in any genetic model. It is worth mentioning that this result is opposite to that of the study published in 2015 [11]; therefore, our study is a necessary update to the literature. Furthermore, the *PRKAA1 rs13361707 C/T* (T-allele) polymorphism can decrease GC risk in the HB and Korean populations, which suggests that the role of this polymorphism in increasing GC susceptibility is limited, and the T-allele may even protect against GC susceptibility, although no evidence supporting this possibility had been found until date. A probable explanation for this phenomenon is that the samples included from Korea were much smaller than those included from China; therefore, even if a significant association exists in the Korean population, its influence was offset by a negative association from the Chinese population and thus no association was detected in the pooled analysis. The same explanation might apply to the association observed in the analysis stratified by source of the control group: a significant inverse relationship was observed for the HB population but not for the pooled population. Besides, complex interactions between several genetic and environmental factors may be involved in cancer development.

In order to better explain the potential function of *PRKAA1 rs13361707 C/T* polymorphism in GC risk, we first used an online gene expression mini database to analyze the expression of *PRKAA1* in both GC and paracancerous tissues. It indicated that the expression of *PRKAA1* was higher in GC tissues. Previously, Varghese et al. [20] also reported that high expression of *PRKAA1* in glioblastoma was correlated with a poor prognosis. Similarly, Huang et al. [21] found that *PRKAA1* had a higher expression in a cervical tumor than in the normal epithelium (*P* < 0.01). Additionally, we found that individuals carrying the TT/TC genotypes of *PRKAA1 rs13361707 C/T* polymorphism had a lower serum expression level than those carrying CC genotypes. This information suggests that *PRKAA1* may be an oncogene. Our analysis, in combination with conclusions reported in the previous meta-analysis [11], suggests that *PRKAA1 rs13361707 C/T* polymorphism, especially the T-allele or TC/TT genotypes, may reduce the expression of *PRKAA1.* Thus, individuals carrying the T-allele or TC/TT genotypes have a lower GC risk.

Meta-analyses have been recognized as an effective method to summarize and review previously published quantitative research to answer a wide variety of clinical questions. However, some limitations in our meta-analysis should be acknowledged. First, the number of published studies included in our meta-analysis remains small for a comprehensive analysis. Second, one of the selected studies included five different case-control sub-analyses in the same paper, possibly with overlap or duplication of some data. This could have influenced the power of our analyses too. Third, gene-gene, gene-environment (including age, sex, family history, environmental factors, disease stage, and lifestyle) and within-gene interactions may modulate GC risk, and therefore should be evaluated in future research. Fourth, all the 10 articles included in our meta-analysis lacked information about *PRKAA1* expression levels classified by gender or stage of the tumor; hence, we could not analyze these factors. We advocate future articles including this information.

In spite of these limitations, our meta-analysis has some advantages. First, the power of our analysis is more than 0.9, lending credibility and persuasiveness to our results. Second, the quality of case-control studies included in the current meta-analysis satisfied our more extensive selection criteria. Third, publication bias was not detected in any of the genetic models, suggesting that the results are relatively stable and generalizable.

In summary, our meta-analysis showed that *PRKAA1 rs13361707 C/T* polymorphism was associated with significantly decreased GC risk in the Korean and HB populations, although no association was found in the pooled populations. Therefore, further well-designed and larger studies, dealing specifically with gene-gene and gene-environment interactions, are warranted.

## MATERIALS AND METHODS

### Identification and eligibility of relevant studies

We conducted searches on the PubMed, Embase, WanFang, and CNKI databases (the last search was conducted on Feb 22, 2017) with the keywords ‘*PRKAA1*’ or ‘protein kinase catalytic subunit alpha-1’ or ‘AMPKa1’, ‘polymorphism’ or ‘variant’ and ‘gastric cancer’ or ‘digestive tract tumor’, without any restriction on language or publication year. Using these terms, a total of 51 articles were retrieved. We also screened references cited in the retrieved articles, and other review articles, by hand.

### Inclusion criteria and exclusion criteria

Studies that were included in our analysis had to meet all of the following criteria: (a) assessment of correlation between GC and *PRKAA1 rs13361707 C/T* polymorphism; (b) case-control design; (c) sufficient numbers for all genotypes (TT, TC, CC) among cases and controls. Additionally, the following exclusion criteria were also used: (a) no control population; (b) no information on genotype frequency; or (c) duplication of previous publications.

### Data extraction

Two authors of this study independently extracted all the data from studies complying with the selection criteria. The following items were collected: first author’s last name, year of publication, country of origin, sample size, total case/control number, subjects of each genotype in cases and controls, source of control (HB or PB), Hardy–Weinberg equilibrium (HWE) of controls, the Newcastle-Ottawa Scale (NOS), GC type, and genotyping methods.

### Quality score assessment

The NOS [22] was used to assess the quality of each study. This measure assesses observational studies on measures of study quality, such as the selection of cases, comparability of populations, and ascertainment of exposure to risks. The NOS ranges from zero (worst) to nine stars (best). Studies with a score of seven stars or greater were considered as high-quality.

### In-silico analysis of PRKAA1 expression

We used the online gene expression mini database from the Zhang Lab of Peking University (http://gemini.cancer-pku.cn/) to analyze the expression of *PRKAA1* in both GC and paracancerous tissues [23]. This database includes RNA expression profiles of 410 GC samples (whose diagnosis was confirmed clinically) and 228 normal samples from the corresponding tissues.

### Statistical analysis

The strength of the association between *PRKAA1 rs13361707 C/T* polymorphism and GC risk in different strata was expressed as odds ratios (ORs) with 95% confidence intervals (CIs). The statistical significance of the ORs was determined with the *Z*-test. Heterogeneity assumption was evaluated by a chi-square-based *Q*-test. When a *P*-value < 0.10 for the *Q*-test indicated a lack of heterogeneity among the studies, the random effects model (DerSimonian-Laird method) was used [24]. Otherwise, the fixed effects model (Mantel-Haenszel method) was chosen [25]. For different *PRKAA1 rs13361707 C/T* genetic variants, we investigated their association with GC risk according to the following models: allelic contrast model (T-allele vs. C-allele), homozygote comparison model (TT vs. CC), heterozygote comparison model (TC vs. CC), dominant genetic model (TT+TC vs. CC) and recessive genetic model (TT vs. TC+CC). Funnel plot asymmetry was assessed using Begg’s test and publication bias was assessed using Egger’s test, *P* < 0.05 was considered statistically significant [26]. The departure of frequencies of *PRKAA1* gene polymorphism from expectation under HWE was assessed in controls by using the Pearson chi-square test; *P* < 0.05 was considered significant. All statistical tests for this meta-analysis were performed using the Stata software (version 11.0; StataCorp LP, College Station, TX). The power of our meta-analysis was calculated by a program named PS: Power and Sample Size Calculation (http://biostat.mc.vanderbilt.edu/wiki/Main/PowerSampleSize#Windows) [27].

### Genotyping methods

Genotyping of *PRKAA1 rs13361707 C/T* was conducted using different techniques in different studies: polymerase chain reaction matrix-assisted laser desorption/ionization time-of-flight mass spectrometry (PCR-MALDI-TOF-MS), high-resolution melting polymerase chain reaction (HRM-PCR), improved multiplex ligase detection reaction (iMLDR), Affymetrix Genome-Wide Human SNP Array 6.4 chips (AGWHSA 6.0 CHIPS), GoldenGate assay (Illumina), Multiplex SNaPshot SNP, TaqMan and kompetitive allele-specific PCR (KASP).

### Study population and genotyping

Two hundred GC patients were newly diagnosed between November 2014 and May 2016 in the Tongzhou People’s Hospital of Nantong (Supplementary Table 2). All GC cases were between 51 and 87 years of age and were diagnosed with the disease within the preceding one year. All cases underwent laparoscopic radical gastrectomy for gastric cancer and were diagnosed by a pathological examination. A 2-ml peripheral blood sample was collected from each patient. Ethics approval was obtained from the ethics committees at the Tongzhou People’s Hospital of Nantong and all samples were collected after each patient provided a written informed consent. The *PRKAA1 rs13361707 C/T* polymorphism was analyzed by a TaqMan assay as described by Li et al. [16].

## SUPPLEMENTARY MATERIALS FIGURES AND TABLES



## References

[1] International Agency for Research on Cancer (2012). Stomach cancer. http://globocan.iarc.fr/Pages/fact_sheets_cancer.aspx.

[2] Sugano K (2015). Screening of gastric cancer in Asia. Best Pract Res Clin Gastroenterol.

[3] Figueiredo C, Constanza Camargo M, Leite M, Fuentes-Pananá EM, Rabkin CS, Machado JC (2017). Pathogenesis of gastric cancer: genetics and molecular classification. Curr Top Microbiol Immunol.

[4] Crew KD, Neugut AI (2006). Epidemiology of gastric cancer. World J Gastroenterol.

[5] Yasui W, Sentani K, Sakamoto N, Anami K, Naito Y, Oue N (2011). Molecular pathology of gastric cancer: research and practice. Pathol Res Pract.

[6] Amieva M1, Peek RM (2016). Pathobiology of helicobacter pylori-induced gastric cancer. Gastroenterology.

[7] Shi Y, Hu Z, Wu C, Dai J, Li H, Dong J, Wang M, Miao X, Zhou Y, Lu F, Zhang H, Hu L, Jiang Y (2011). A genome-wide association study identifies new susceptibility loci for non-cardia gastric cancer at3q13.31 and 5p13.1. Nat Genet.

[8] Daskalopoulos EP, Dufeys C, Bertrand L, Beauloye C, Horman S (2016). AMPK in cardiac fibrosis and repair: actions beyond metabolic regulation. J Mol Cell Cardiol.

[9] Xu L, Ash JD (2016). The role of AMPK pathway in neuroprotection. Adv Exp Med Biol.

[10] Park C, Jeong JS, Jeong JW, Kim YJ, Jung YK, Go GB, Kim SO, Kim GY, Hong SH, Yoo YH, Choi YH (2016). Ethanol extract of Kalopanax septemlobus leaf induces caspase-dependent apoptosis associated with activation of AMPK in human hepatocellular carcinoma cells. Int J Oncol.

[11] Qiu LX, He J, Cheng L, Zhou F, Wang MY, Sun MH, Zhou XY, Li J, Guo WJ, Wang YN, Yang YJ, Wang JC, Jin L (2015). Genetic variant of PRKAA1 and gastric cancer risk in an eastern Chinese population. Oncotarget.

[12] Eom SY, Hong SM, Yim DH, Kwon HJ, Kim DH, Yun HY, Song YJ, Youn SJ, Hyun T, Park JS, Kim BS, Kim YD, Kim H (2016). Additive interactions between PRKAA1 polymorphisms and Helicobacter pylori CagA infectionassociated with gastric cancer risk in Koreans. Cancer Med.

[13] Cai M, Dai S, Chen W, Xia C, Lu L, Dai S, Qi J, Wang M, Wang M, Zhou L, Lei F, Zuo T, Zeng H (2017). Environmental factors, seven GWAS-identified susceptibility loci, and risk of gastric cancer and itsprecursors in a Chinese population. Cancer Med.

[14] Zhang YJ, Zhang CY, Yu XJ, Xu XN, Wang LL, Geng CX, Dong QJ (2016). The correlation between polymorphisms of PRKAA1 and UNC5CL and susceptibility to gastric cancer. Chinese J Clin Doctors.

[15] Song HR, Kim HN, Kweon SS, Choi JS, Shim HJ, Cho SH, Chung IJ, Park YK, Kim SH, Choi YD, Joo KW, Shin MH (2013). Genetic variations in the PRKAA1 and ZBTB20 genes and gastric cancer susceptibility in a Koreanpopulation. Mol Carcinog.

[16] Li M, Huang L, Qiu H, Fu Q, Li W, Yu Q, Sun L, Zhang L, Hu G, Hu J, Yuan X (2013). Helicobacter pylori infection synergizes with three inflammation-related genetic variants in theGWASs to increase risk of gastric cancer in a Chinese population. PLoS One.

[17] Dong Y, Chen J, Chen Z, Tian C, Lu H, Ruan J, Yang W (2015). Evaluating the association of eight polymorphisms with cancer susceptibility in a Han Chinese population. PLoS One.

[18] Kim YD, Yim DH, Eom SY, Moon SI, Yun HY, Song YJ, Youn SJ, Hyun T, Park JS, Kim BS, Lee JY, Won HK, Kim H (2014). Risk of gastric cancer is associated with PRKAA1 gene polymorphisms in Koreans. World J Gastroenterol.

[19] Wu D (2014). Association of Helicobacter pylori infection and polymorphisms pf PSCA, PLCE1 and PRKAA1 with gastric cancer susceptibility. http://kns.cnki.net/KCMS/detail/detail.aspx?dbcode=CMFD&dbname=CMFD201402&filename=1014301504.nh&v=MTk3Mzc3ckJWRjI2R3JDNEg5VE1xNUViUElSOGVYMUx1eFlTN0RoMVQzcVRyV00xRnJDVVJMMmZaZVJzRkNya1c=.

[20] Varghese RT, Liang Y, Guan T, Franck CT, Kelly DF, Sheng Z (2016). Survival kinase genes present prognostic significance in glioblastoma. Oncotarget.

[21] Huang FY, Chiu PM, Tam KF, Kwok YK, Lau ET, Tang MH, Ng TY, Liu VW, Cheung AN, Ngan HY (2006). Semi-quantitative fluorescent PCR analysis identifies PRKAA1 on chromosome 5 as a potential candidate cancer gene of cervical cancer. Gynecol Oncol.

[22] Wells GA, Shea B, O'Connell D, Peterson J, Welch V, Losos M, Tugwell P The Newcastle–Ottawa Scale (NOS) for assessing the quality of nonrandomised studies in meta-analyses. http://www.ohri.ca/programs/clinical_epidemiology/oxford.asp.

[23] Tang Z, Li C, Zhang K, Yang M, Hu X (2017). GE-mini: a mobile APP for large-scale gene expression visualization. Bioinformatics.

[24] Mantel N, Haenszel W (1959). Statistical aspects of the analysis of data from retrospective studies of disease. J Natl Cancer Inst.

[25] DerSimonian R, Laird N (1986). Meta-analysis in clinical trials. Control. Clin. Trials.

[26] Hayashino Y, Noguchi Y, Fukui T (2005). Systematic evaluation and comparison of statistical tests for publication bias. J Epidemiol.

[27] Dupont WD, Plummer WD (1998). Power and sample size calculations for studies involving linear regression. Control Clin Trials.

