# Supplementary material for: Correlation between protein kinase catalytic subunit alpha-1 gene rs13361707 polymorphism and gastric cancer susceptibility in asian populations
Source: Oncotarget. 2017 Jul 18;8(40):68354–64. doi: 10.18632/oncotarget.19355 (PMC5620262; doi:10.18632/oncotarget.19355)
Supplement: Supplementary file 1 [file oncotarget-08-68354-s001.pdf]

Correlation between protein kinase catalytic subunit alpha-1 gene rs13361707 polymorphism and gastric cancer susceptibility in asian populations

SUPPLEMENTARY MATERIALS

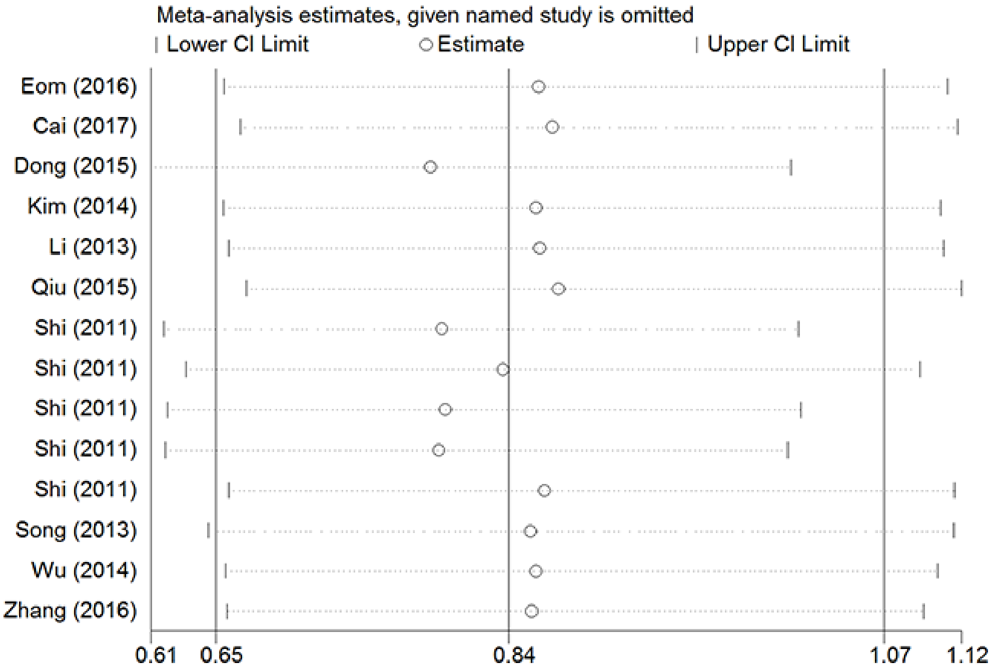

Supplementary Figure 1: Sensitivity analysis between *PRKAA1 rs13361707 C/T* polymorphism and GC risk (TT *vs.* TC+CC).

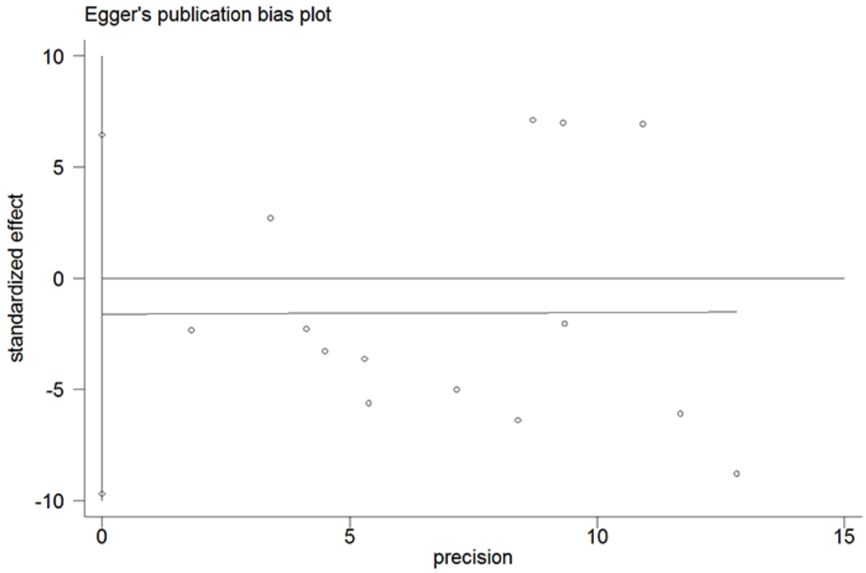

Supplementary Figure 2: Egger's publication bias plot (TA *vs.* AA).

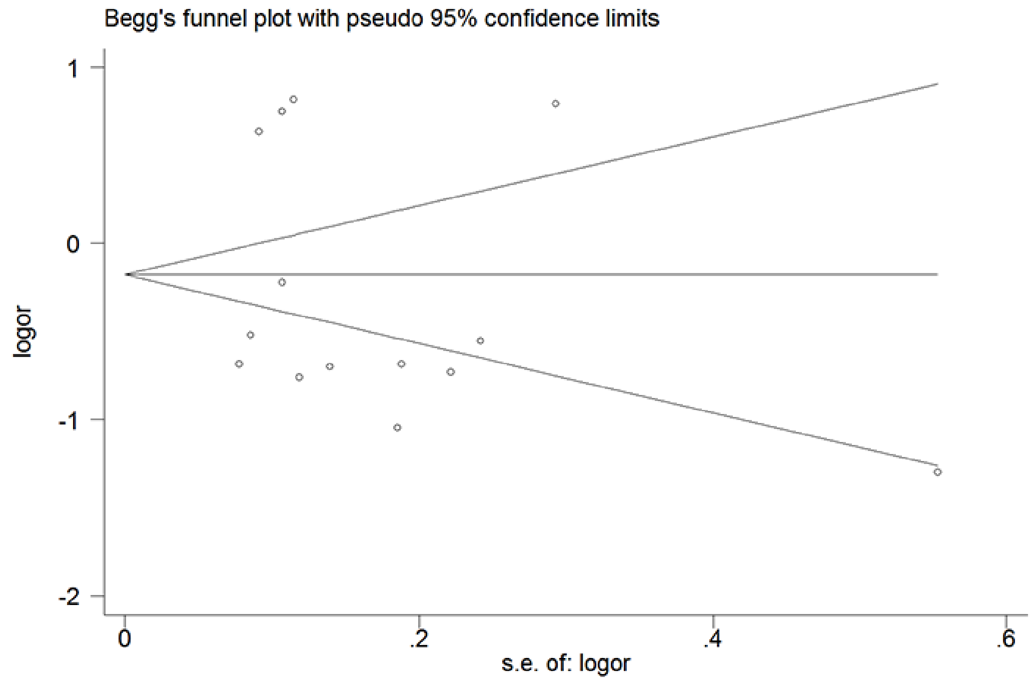

Supplementary Figure 3: Begg's funnel plot for publication bias test (TA *vs.* AA).

Supplementary Table 1: Publication bias tests (Begg's funnel plot and Egger's test for publication bias test) for *PRKAA1 gene* rs13361707 polymorphism

	Egger's test					Begg's test	
**Genetic type**	**Coefficient**	**Standard error**	***t***	***P* value**	**95% CI of intercept**	***z***	***P* value**
**T-allele vs. C-allele**	-3.669	7.539	-0.49	0.635	(-20.097, 12.758)	0.44	0.661
**TC vs. CC**	-1.092	4.212	-0.26	0.8	(-10.269, 8.085)	0.77	0.443
**TT vs. CC**	-1.632	3.711	-0.44	0.668	(-9.717, 6.453)	0.55	0.584
**TT+TC vs. CC**	-1.112	4.465	-0.25	0.808	(-10.841, 8.616)	0.66	8.616
**TT vs. TC+CC**	-3.154	4.631	-0.68	0.509	(-13.244, 6.936)	0.66	0.511

Supplementary Table 2: Basic characteristics of GC patients in our present research

Features	GC patients
n	200
Age, n%	
<60	106
≥60	94
Sex, n%	
Male	112
Female	88
Smoking, n (%)	
Ever	102
Never	98
Alcohol drinking, n (%)	
Ever	116
Never	84
H. pylori infection, n (%)	
Positive	87
Negative	113
Tumor site, n (%)	
Cardia	23
Noncardia	177
Stage, n (%)	
Stage I/II	137
Stage III/IV	63
